# Personalized bioconversion of *Panax notoginseng* saponins mediated by gut microbiota between two different diet-pattern healthy subjects

**DOI:** 10.1186/s13020-021-00476-5

**Published:** 2021-07-23

**Authors:** Li Wang, Man-Yun Chen, Li Shao, Wei Zhang, Xiang-Ping Li, Wei-Hua Huang

**Affiliations:** 1 Department of Clinical Pharmacology, Xiangya Hospital, Central South University, Changsha, 410008 China; 2 Institute of Clinical Pharmacology, Hunan Key Laboratory of Pharmacogenetics, Central South University, Changsha, 410078 China; 3 National Clinical Research Center for Geriatric Disorders, Xiangya Hospital, Central South University, Xiangya Road 110, Changsha, 410008 China; 4 Department of Pharmacognosy, School of Pharmacy, Hunan University of Chinese Medicine, Changsha, 410128 Hunan China; 5 Department of Pharmacy, Xiangya Hospital, Central South University, Changsha, 410008 China; 6 NHC Key Laboratory of Birth Defect for Research and Prevention, Hunan Provincial Maternal and Child Health Care Hospital, Hunan, 410008 China

**Keywords:** *Panax notoginseng* saponins, Gut microbiota, 16S rRNA gene sequencing, Metabolic variation, Biotransformation

## Abstract

**Background:**

*Panax notoginseng* saponins (PNS) as the main effective substances from *P. notoginseng* with low bioavailability could be bio-converted by human gut microbiota. In our previous study, PNS metabolic variations mediated by gut microbiota have been observed between high fat, high protein (HF-HP) and low fat, plant fiber-rich (LF-PF) dietary subjects. In this study, we aimed to correspondingly characterize the relationship between distinct gut microbial species and PNS metabolites.

**Methods:**

Gut microbiota were collected from HF-HP and LF-PF dietary healthy adults and profiled by 16S rRNA gene sequencing. PNS were incubated with gut microbiota in vitro. A LC–MS/MS method was developed to quantify the five main metabolites yields including ginsenoside F_1_ (GF_1_), ginsenoside Rh_2_ (GRh_2_), ginsenoside compound K (GC-K), protopanaxatriol (PPT) and protopanaxadiol (PPD). The selected microbial species, *Bifidobacterium adolescentis* and *Lactobacillus rhamnosus*, were employed to metabolize PNS for the corresponding metabolites.

**Results:**

The five main metabolites were significantly different between the two diet groups. Compared with HF-HP group, the microbial genus *Blautia*, *Bifidobacterium, Clostridium, Corynebacterium, Dorea, Enhydrobacter*, *Lactobacillus, Roseburia*, *Ruminococcus, SMB53, Streptococcus, Treponema* and *Weissella* were enriched in LF-PF group, while *Phascolarctobacterium* and *Oscillospira* were relatively decreased. Furthermore, Spearman’s correlative analysis revealed gut microbials enriched in LF-PF and HF-HP groups were positively and negatively associated with the five metabolites, respectively.

**Conclusions:**

Our data showed gut microbiota diversity led to the personalized bioconversion of PNS.

**Graphic Abstract:**

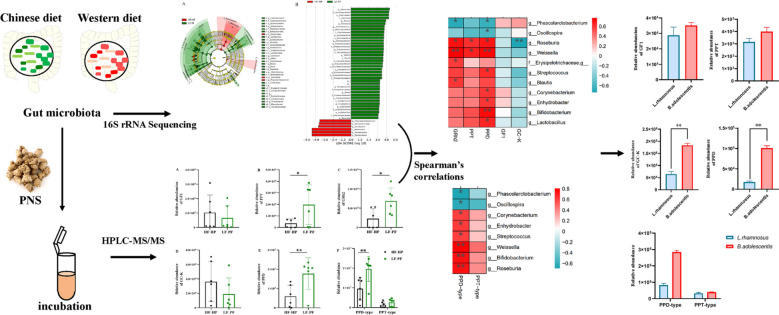

**Supplementary Information:**

The online version contains supplementary material available at 10.1186/s13020-021-00476-5.

## Highlights

- *Panax notoginseng* saponins could be biotransformed to generate five main metabolites, including GF_1_, GRh_2_, GC-K, PPT and PPD, by human gut microbiota.
- Gut microbiota profiles were significantly different in high protein, high fat and low fat, plant fiber-rich diet-pattern groups.
- Correlation analysis revealed potential relationships between metabolites and gut microbial species.
- *Bifidobacterium adolescentis* and *Lactobacillus rhamnosus* were selected as a representative species to metabolize PNS for the concerned metabolites.

**Supplementary Information:**

The online version contains supplementary material available at 10.1186/s13020-021-00476-5.

## Introduction

*Panax notoginseng* saponins (PNS) as the main health-beneficial components in *P. notoginseng* are limited with low bioavailability due to their poor membrane permeability [1]. However, after orally administrated, PNS inevitably interact with gut microbiota in gastrointestinal tract, which could be bio-converted to be novel bioactive metabolites [2, 3]. For PNS metabolism, gut microbiota are mainly involved in deglycosylation reaction with hydrolyzing the oligosaccharide chains, which are catalyzed by the microbial *β*-glycosidases [4, 5]. Recently, PNS bioconversion mediated by gut microbiota has been reported to reveal the metabolic profile of PNS [4, 6]. However, significant variations of PNS metabolism were discovered between two different dietary-driven human gut microbiota groups in our previous study [7]. Undoubtedly, the metabolic variations will alter the pharmacological effects of PNS. Due to the complex gut microbiome characterized with different xenobiotic-metabolizing enzymes, the metabolism profiles of PNS still remain largely elusive.

Besides the intrinsic host genetic makeup, gut microbiota possess a dynamic balance with external environment exposure, such as nutritional state and disease status. The most effective determining factor is the daily dietary pattern of healthy subjects, which could modulate the profile of gut microbiota [8]. Obviously, the personalized gut microbial phylotypes will lead to the metabolic variations of PNS. Therefore, the inter-diversity of gut microbiota between HF-HP and LF-PF groups was the focal point in this study, instead of focusing on the intra-group differences.

The key development of high-throughput sequencing technology as a perspective application makes it possible to insight into the composition, diversity, even the gene functions of gut microbiome through the analysis of 16S rRNA sequencing or whole-genome shotgun sequencing [9, 10]. Herein, to clarify the metabolism variations of PNS mediated by gut microbiota, it was necessary to elucidate the relationship between PNS metabolites and gut microbials driven by different diet patterns.

In the present study, gut microbiota were randomly collected from six high fat, high protein (HF-HP) and six low fat, plant fiber-rich (LF-PF) dietary-pattern healthy subjects, respectively. The V3-V4 region of 16S rRNA gene was sequenced on an Illumina HiSeq 2500. The main metabolites of PNS (GF_1_, GRh_2_, GCK, PPT and PPD) were relatively quantified by a high performance liquid chromatography-electrospray ionization tandem mass spectrometry (HPLC–ESI–MS/MS). Alpha- and beta-diversities were employed to evaluate the richness and evenness of gut microbiome. Both of Operational Taxonomic Units (OTUs) and predictive functional profiles of gut microbials were used to represent the inter-difference between the two groups. Moreover, *Bifidobacterium adolescentis* and *Lactobacillus rhamnosus* were selected as the representative species of *Bifidobacterium* and *Lactobacillus* to verify the results. Altogether, our data indicated that the composition and diversity of gut microbiota could be modulated by different diets, which led to metabolism variations of PNS.

## Materials and methods

### Chemicals and reagents

General anaerobic medium (GAM) broth was obtained from Nissui Pharmaceutical Inc. (Tokyo, Japan). Leibovitz’s L-15 medium was purchased from Life Technologies Co. (Grand Island, NY, USA). Brain heart infusion (BHI) broth was manufactured from Oxoid Ltd. (Basingstoke, England). Fetal bovine serum (FBS) was purchased from Gibco (Gaithersburg, MD, USA). HPLC-grade acetonitrile (ACN) was purchased from Merck (Darmstadt, Germany). Deionized water (18 MΩ cm^−1^) was purified using a Milli-Q Ultrapure water system (Milford, MA, USA). Ginsenoside F_1_, GRh_2_, and PPT were bought from Baoji Herbest Bio-Tech Co., Ltd. (Shaanxi, China). GC-K, PPD and digoxin (the internal standard, IS) were provided by Chengdu Push Bio-technology Co., Ltd. (Sichuan, China). The purity of all compounds was determined by HPLC (≥ 98%), and their chemical structures were shown in Additional file 1: Fig. S1.

Bacteria genomic DNA extraction kit was purchased from Omega Bio-tek (Norcross, GA, USA). Mixture Polymerase Chain Reaction (PCR) product purification kit was purchased from Qiagen (Hilden, Germany). Sequencing library generation kit was purchased from Illumina (San Diego, USA).

### Sample collection and gut microbiota preparation

Stool samples were collected from HF-HP and LF-PF dietary-pattern healthy subjects. Inclusion criteria were set as, (i) age between 20 and 25 years; (ii) body mass index (BMI) between 19 and 24 kg/m^2^; (iii) absence of systemic and metabolic disease; (iv) no use of alcohol and tobacco; and (v) stable diet pattern in the last one year, referring to HF-HP and LF-PF diets. Exclusion criteria were defined as, (i) history of any antibiotics or probiotics medications in the last three months; (ii) history of drug allergies and highly sensitive to environmental; and (iii) mental illness rendering the participants unable to understand the nature, scope, and possible consequences of the study. The energy intake used to differ HF-HP and LF-PF diet patterns was calculated by the ratio of protein, carbohydrate and fat in different types of foods. The low-fat (fat < 20% energy) and high-fat (fat > 35% energy) diet were relatively defined and recommended by the World Health Organization and the UN Food and Agriculture Organization. Energy content, macronutrient composition, and Fiber content of the HF-HP and LF-PF diets were shown in Additional file 2: Table S3. All individuals provided written informed consent prior to participating in the study.

According to our previous study [7], 1 g of fresh fecal sample was suspended in 20 mL of cold physiological saline and then centrifuged to collect the resultant fecal supernatant. The precipitation was re-suspended with Leibovitz’s L-15 medium containing glycerol as the gut microbiota solution stored in − 80 ℃ freeze.

### PNS preparation and incubation

The air-dried root of *P. notoginseng* was purchased from Wenshan city (Yunnan, China) and extracted by heat-refluxing with 70% ethanol to obtain the *P. notoginseng* extract. The detailed information about *P. notoginseng* and PNS extraction were described in our former publication [7].

Gut microbiota stock was activated with mGAM broth and then centrifuged to collect the gut microbiota precipitation. Leibovitz’s L-15 medium was added to re-suspend the precipitate as the gut microbiota work solution. Gut microbiota work solution, *P. notoginseng* extract stock solution in dimethyl sulphoxide (DMSO) and Leibovitz’s L-15 medium were mixed as incubation system incubated at 37 ℃ for 48 h. The reaction mixtures were successively extracted by ethyl acetate and n-butanol, and evaporated under nitrogen. At last, the samples were reconstituted with methanol before subjected to HPLC analysis. The specific plan was described in our previous study[7]. *L. rhamnosus* and *B. adolescentis* were purchased from American Type Culture Collection (ATCC). *B. adolescentis* and *L. rhamnosus* were cultured in mGAM and Brain Heart Infusion Medium(BHI) for 24 h before incubating 48 h with PNS at 37 ℃. The reaction mixtures were extracted as the same with above-mentioned methods. PNS (175 μg/ml) was respectively added into the culture system of *B. adolescentis* and *L. rhamnosus* to detect their growth dynamics by comparing with combined antibiotics (ampicillin, metronidazole, vancomycin and neomycin; 20 μΜ, resp.)[10].

### Relative quantification of metabolites by HPLC–MS

The PNS metabolites biotransformed by gut microbiota were quantified by comparing the five main metabolites between LF-PF and HF-HP diet groups on HPLC–ESI–MS/MS system, which consisted of a SHIMADZU Nexera X2 HPLC system (SHIMADZU, Tokyo, Japan) and AB SCIEX Triple Quad TM 6500 mass spectrometers equipped with electrospray ionization (AB SCIEX, CA, USA). According to the methods[11], the chromatographic separation was performed on a Phenomenex Luna C18 (2) column (150 × 2.0 mm, id, 5 μm) with a gradient elution of 0.1% formic acid in water (A) and ACN (B) at a flow rate of 0.3 mL/min. The gradient profile was optimized as below, 0–2 min: 35%–65% B, 2–6 min: 65%–70% B, 6–7 min: 70%–72% B, 7–7.5 min: 72%–85% B, 7.5–8 min: 85%–85% B, 8–11 min: 85%–95% B,11–12 min: 95%–100% B. The injection volume was 2 μL with the temperature of column set at 40 ℃. The mass spectrometer parameters were optimized in positive ion mode, spray voltage, 4500 V; temperature, 350 ℃; collision gas, 10 psi; curtain gas, 35 psi; ion source gas 1, 55 psi; ion source gas 2, 50 psi.

### Method validation

Five metabolites including GF_1_, Rh_2_, GC-K, PPD, PPT and digoxin (IS) were mixed and dissolved in methanol to validate this method. For intra-day precision, the standards were analyzed three times within one day, while they were determined in triplicate for three successive days for inter-day precision. Relative standard deviations (RSDs) were calculated to evaluate the variations. Selectivity was investigated by comparing the spectra of blank human gut microbiota with/without IS or with analytes and IS to exclude the peaks of endogenous components in the incubation system.

### 16S rRNA gene sequencing and data analysis

Microbial genomic DNA was extracted from each sample and stored in − 20 ℃ using the Qiagen QIAamp DNA Stool Mini Kit (Qiagen). DNA concentration was estimated using a nanodrop instrument (Thermo Scientific), and the purity of DNA was monitored on 1% agarose gels. Subsequently, DNA was diluted to 1 ng/μL using sterile water. The variable region V3-V4 of the bacteria 16S rRNA gene from each sample were amplified using the bacterial universal primer 338F 5′-ACTCCTACGGGAGGCAGCAG-3′ and 806R 5′-barcode GGACTACHVGGGTWTCTAAT-3′, while barcode was a six-base unique sequence to each sample.

The PCR products of the same sample were mixed firstly, which were extracted from 2% agarose gels and purified by the AxyPrep DNA Gel Extraction Kit (Axygen Biosciences, Union City, CA, U.S.) according to the manufacturer’s instructions. Referring to the preliminary quantitative results of electrophoresis, QuantiFluor™ -ST blue fluorescence quantitative system was used to detect and quantify the PCR products. Purified amplicons were pooled in equimolar and sequenced (2 × 250) on an Illumina MiSeq platform according to the standard protocols. The 16S rRNA gene sequencing of gut microbiota was completed by Shanghai Biotechnology Corporation.

Sequence alignment, operational taxonomic units (OTUs), clustering, phylogenetic and taxonomic profiling and the analysis of beta diversity were performed with the Quantitative Insights into Microbial Ecology (QIIME2) open source software package. Differential genera bacteria were identified using LEfSe analysis. We used PICRUSt to predict the metabolic functions of gut microbiota. A heat map was constructed with a cluster tree using the Microeco bioinformatics cloud (https://www.bioincloud.tech).

### Statistical analysis

Spearman’s correlation analysis and Student’s *t*-test were performed using SPSS software (Version 23). Significant differences were set as * *p* < 0.05 and ** *p* < 0.01 or *q* < 0.01.

## Results

### Method validation

The intra-day variations (RSDs, n = 12) of GF_1_, GRh_2_, PPD, PPT and GC-K were 8.77%, 6.67%, 8.37%, 8.63% and 6.42%, respectively, and the inter-day RSDs were 13.4%, 13.3%, 13.3%, 12.9% and 12.1%, respectively. The data indicated that the employed method was accurate and precise. This method displayed a good selectivity for the detection of all analytes. There was no significant endogenous interference in the chromatograms of analytes and IS in all blank human gut microbiota samples. Baseline separation has been achieved between IS and analytes (Additional file 1: Figs. S2 and S3).

### Biotransformation of PNS mediated by gut microbiota

**Fig. 1:**
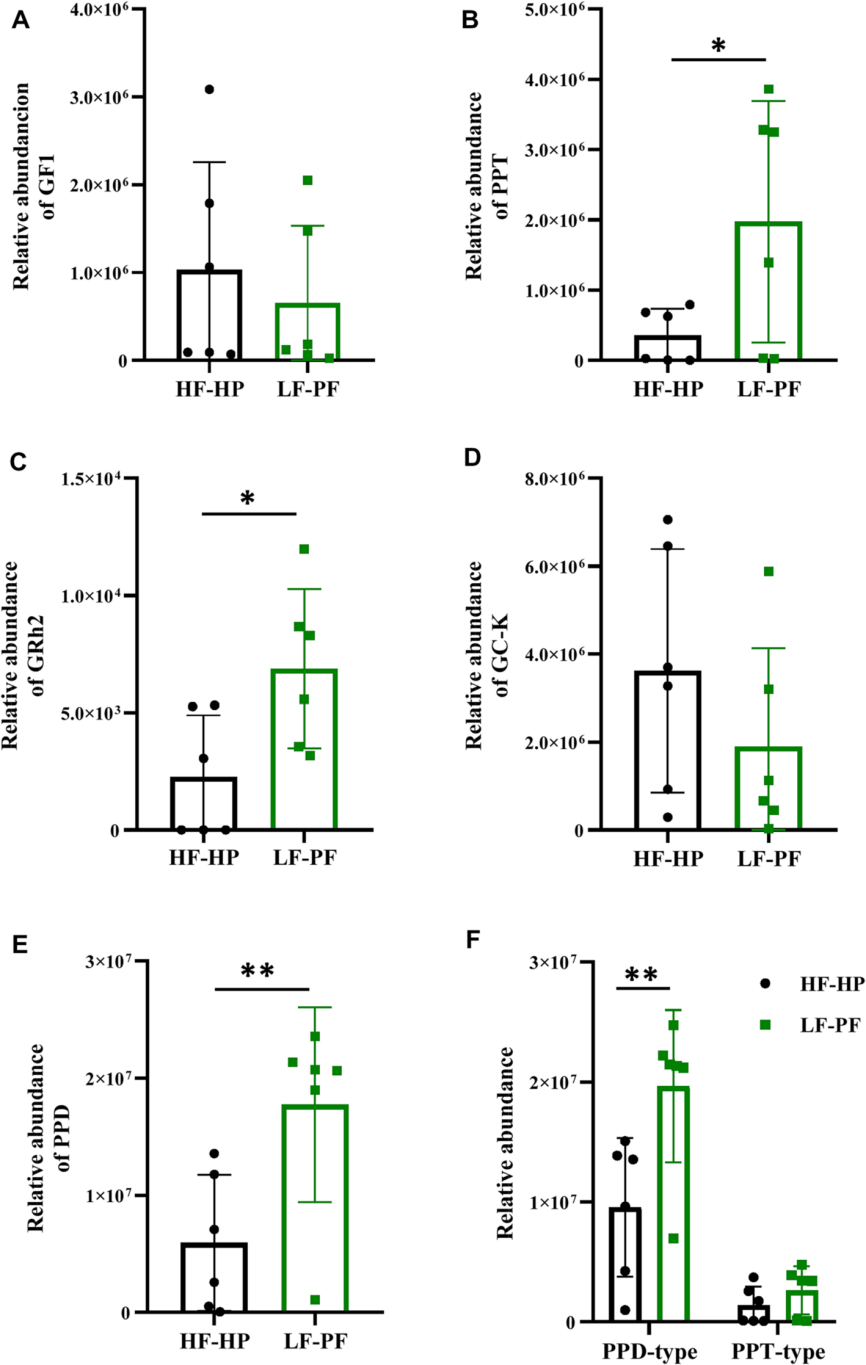
The relative quantities of five main *P. notoginseng* saponins metabolites. The relative quantities of GF_1_ (**A**), PPT (**B**), GRh_2_ (**C**), GC-K (**D**), PPD (**E**), PPT- and PPD- type secondary ginsenosides (**F**) in LF-PF and HF-HP groups

In our previous study [7], forty-five metabolites of PNS were identified by HPLC–DAD-QTOF-MS/MS after incubating PNS with human gut microbiota in vitro. To evaluate the metabolic variations between HF-HP and LF-PF groups, five main metabolites were relatively quantified and compared with each other from different healthy subjects (Fig. 1A–E). The results showed significant differences in the relative quantities of GRh_2_, PPT and PPD between the two groups (*p* < 0.05). Compared with HF-HP group, GRh_2_, PPT and PPD were much more in LF-PF group, but GF_1_ and GC-K were much less. Furthermore, the abundances of PPD-type secondary ginsenosides were significantly higher than PPT-type secondary ginsenosides in LF-PF group, who had stronger ability to metabolize PPD-type ginsenosides (Fig. 1F). Moreover, the considerable variations of metabolites abundance also occurred even within the same group.

### Alpha- and beta-diversity of gut microbiota

**Fig. 2:**
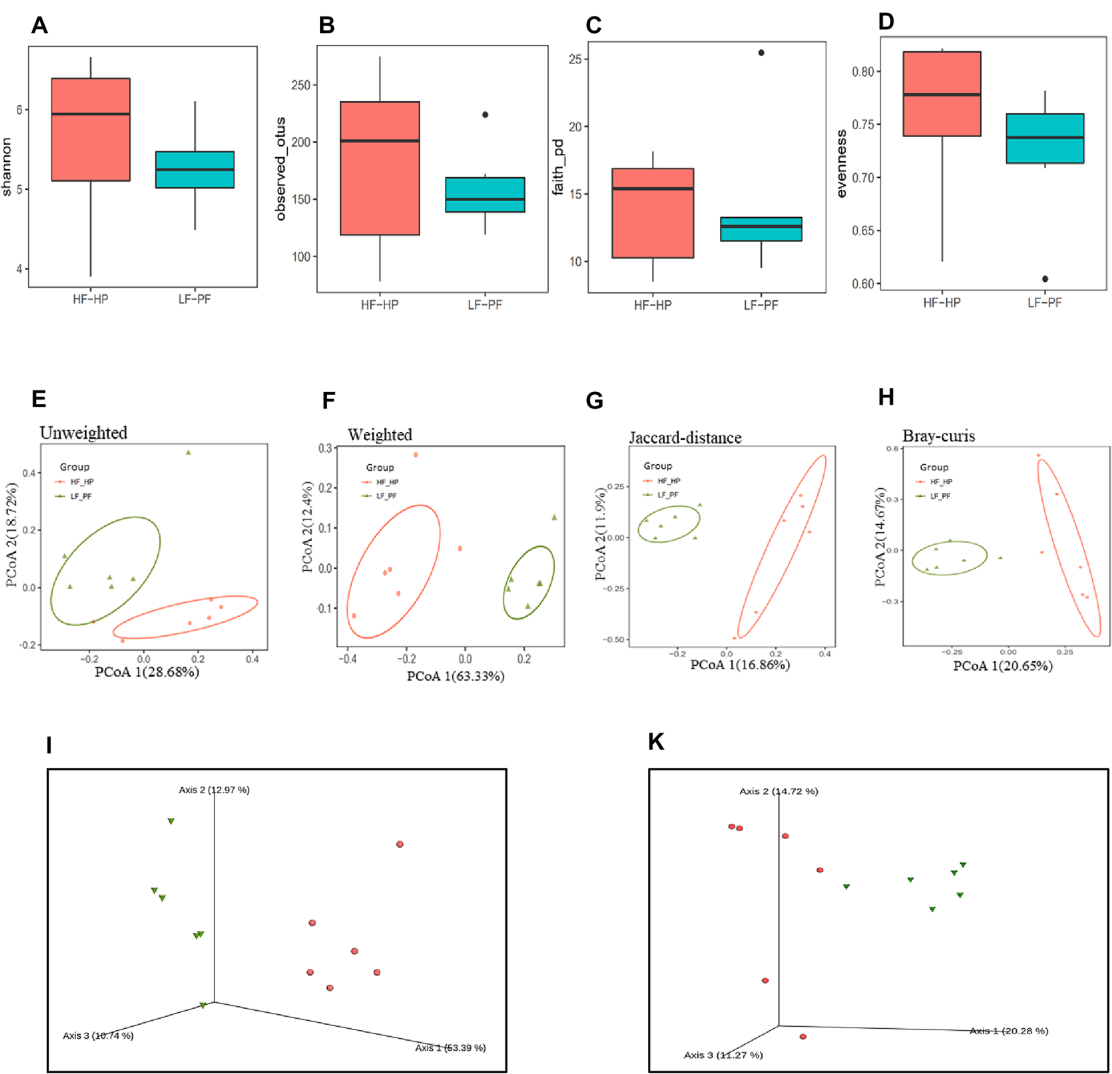
The alpha- and beta-diversity of the gut microbiota collected from LF-PF and HF-HP diet groups. Alpha-diversity analysis based on Shannon index (**A**), Observed OTUs (**B**), Faith’s index (**C**) and Evenness index (**D**). Beta-diversity analysis based on Unweighted unifrac distance (**E**), Weighted unifrac distance (**F** and **I**), Jaccard distance (**G**) and Bray–Curtis distance (**H** and **K**). Each point represented a sample with different color. Red and green curves were respectively composed of HF-HF and HF-HP diet healthy subjects

As shown in the rarefaction curves (Additional file 1: Fig. S5), the sequence reads were enough to carry out species richness and evenness estimates. Compared with LF-PF group, HF-HP group had higher richness for gut microbial diversity (Fig. 2A). No significant alpha-diversity metrics was found between the two diet groups. Other richness estimators, such as Observed OTUs and Faith’s index, also revealed no statistically differences at OTU level (97%) (Fig. 2B and C). Evenness index (Fig. 2D) showed no discrimination between the two groups, indicating that the evenness of species was comparable between the two groups. Beta-diversity was evaluated by principal component analysis (PCoA). As shown in Fig. 2E, F, despite large inherent individual differences in gut microbiota appeared even within the same diet group, the results unambiguously supported that the PCoA plots could be divided into two clusters. Alpha- and beta- diversity of gut microbiota were significantly different between LF-PF and HF-HP groups.

### Taxonomic differences of gut microbiota

**Fig. 3:**
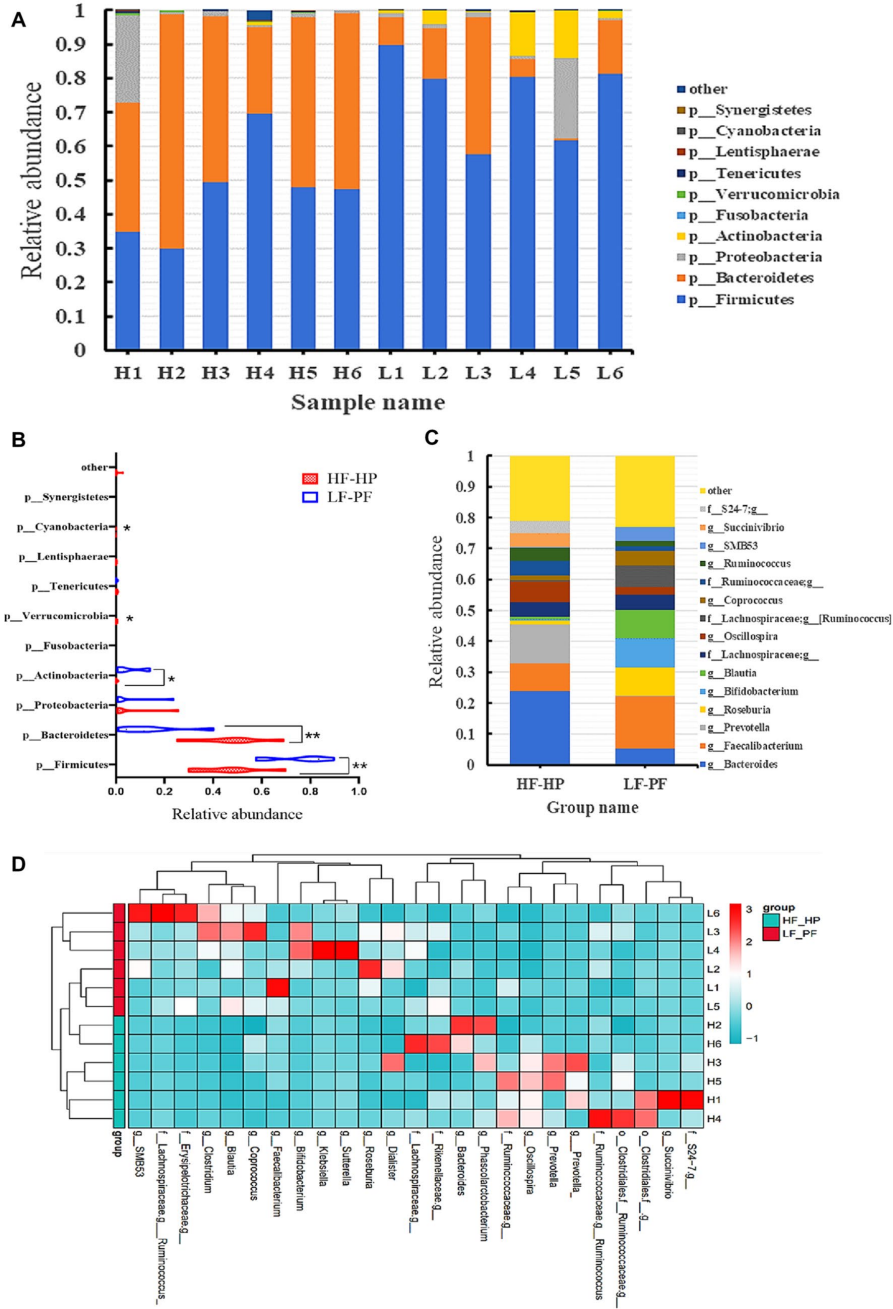
Microbial signatures of the gut microbiota in different diet groups. Phyla-level microbial classification of individual stool samples (**A**) and the two diet groups (**B**); Genus-level microbial classification of the two diet groups (**C**); A clustering analysis based on the abundance of the top 25 features (**D**)

We obtained an average of 1374 features per DNA sample extracted from fecal samples. Taxonomy-based comparison of gut microbiota was performed to elucidate the overall community structure of gut microbiota on phyla and genus levels between the two groups. Relative abundances of the top ten gut microbials at phyla were shown in Fig. 3A and B, respectively. Compared with LF-PF group, the phyla Bacteroidetes, Cyanobacteria, Lentisphaerae, Proteobacteria, Spirochaetes, Verrucomicrobia and Tenericutes were enriched in HF-HP group, while Actinobacteria, Firmicutes and Fusobacteria were decreased relatively. At the genus level, the top fifteen gut microbials were shown in Fig. 3C. Compared with HF-HP group, the genus *Blautia*, *Bifidobacterium*, *Roseburia*, *Ruminococcus* and *SMB53* were enriched in LF-PF group, while *Oscillospira* was relatively decreased. A Clustering analysis based on the abundances of the top 25 features were transformed into a heat map, which revealed two main clusters (Fig. 3D). The results displayed the diversities of gut microbiota could be modulated by diet patterns.

**Fig. 4:**
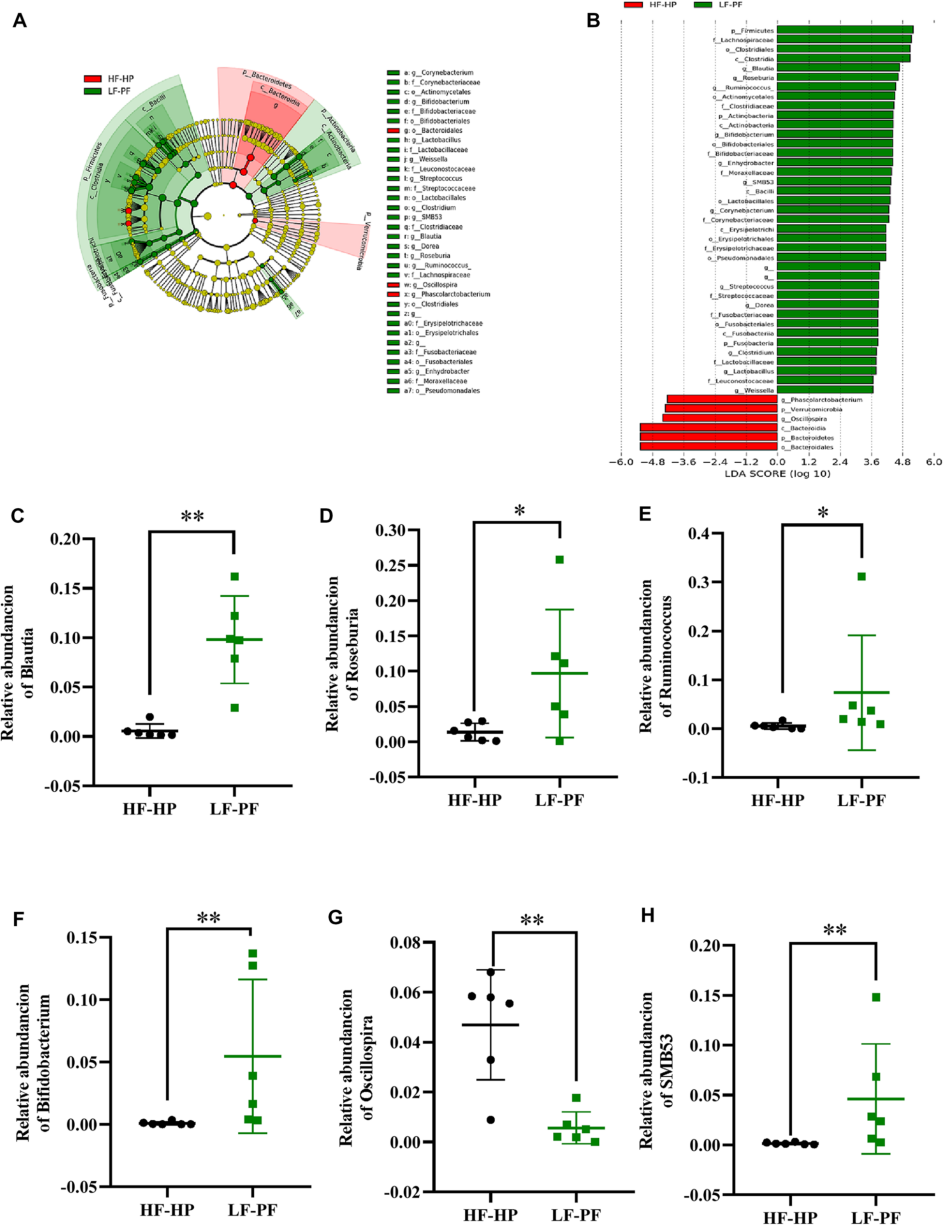
Taxonomic differences of gut microbiota between LF-PF and HF-HP diet groups. Taxonomic cladogram with LEfSe for data analysis and visualization. Taxa with enriched levels in HF-HP and LF-PF groups (**A**); LDA scores (> 2) observed for individual taxa (**B**). Relative abundance of *Blautia, Bifidobacterium, Roseburia, Ruminococcus, SMB53* and *Oscillospira* between the two groups (**C**–**H**)

We also used the linear discriminative analysis (LDA) effect size (LEfSe) biomarker discovery tool to identify taxonomic differences between two diet-pattern groups (Fig. 4A and B). In genus level, the biomarkers for the HF-HP cluster were *Oscillospira* and *Phascolarctobacterium*, while the biomarkers of LF-PF cluster were *Bifidobacterium*, *Blautia*, *Clostridium*, *Corynebacterium*, *Dorea*, *Enhydrobacter*, *Lactobacillus*, *Roseburia*, *Ruminococcus, SMB53, Streptococcus, Treponema* and *Weissella*. Moreover, *Blautia*, *Bifidobacterium*, *Roseburia*, *Ruminococcus, SMB53* and *Oscillospira* (Fig. 4C–H) were different with higher abundance in the genus level. These biomarkers presented high LDA scores (LDA > 2) and were enriched in Firmicutes and Actinobacteria phylum (Table 1).

**Table 1:** Taxonomic differences of gut microbiota between LF-PF and HF-HP diet groups

No./ Tax	Kingdom	Phylum	Class	Order	Family	Genus
1	*Bacteria*	Firmicutes	Clostridia	Clostridiales	Lachnospiraceae	*Blautia*
2	*Bacteria*	Firmicutes	Clostridia	Clostridiales	Lachnospiraceae	*Roseburia*
3	*Bacteria*	Firmicutes	Clostridia	Clostridiales	Lachnospiraceae	*[Ruminococcus]*
4	*Bacteria*	Actinobacteria	Actinobacteria	Bifidobacteriales	Bifidobacteriaceae	*Bifidobacterium*
5	*Bacteria*	Firmicutes	Clostridia	Clostridiales	Ruminococcaceae	*Oscillospira*
6	*Bacteria*	Firmicutes	Clostridia	Clostridiales	Clostridiaceae	*SMB53*
7	*Bacteria*	Firmicutes	Clostridia	Clostridiales	Veillonellaceae	*Phascolarctobacterium*
8	*Bacteria*	Firmicutes	Clostridia	Clostridiales	Lachnospiraceae	*Dorea*
9	*Bacteria*	Firmicutes	Clostridia	Clostridiales	Clostridiaceae	*Clostridium*
10	*Bacteria*	Firmicutes	Erysipelotrichi	Erysipelotrichales	Erysipelotrichaceae	*__*
11	*Bacteria*	Firmicutes	Bacilli	Lactobacillales	Streptococcaceae	*Streptococcus*
12	*Bacteria*	Fusobacteria	Fusobacteriia	Fusobacteriales	Fusobacteriaceae	*__*
13	*Bacteria*	Firmicutes	Bacilli	Lactobacillales	Lactobacillaceae	*Lactobacillus*
14	*Bacteria*	Firmicutes	Bacilli	Lactobacillales	Leuconostocaceae	*Weissella*
15	*Bacteria*	Actinobacteria	Actinobacteria	Actinomycetales	Corynebacteriaceae	*Corynebacterium*
16	*Bacteria*	Proteobacteria	Gammaproteobacteria	Pseudomonadales	Moraxellaceae	*Enhydrobacter*

The "_" in the table indicates unannotated species

Finally, we used PICRUSt (Additional file 1: Fig. S6) to predict the metabolic function spectra of gut microbials. A total of 328 KEGG functional pathways were enriched, and 57 functional pathways were statistically different between the two groups (p < 0.05). Among them, 15 pathways were significantly enriched in the LF-PF group, such as ABC transporters, phosphotransferase system, porphyrin and chlorophyll metabolism, with 42 pathways in the HF-HP group, such as pyrimidine metabolism, lipopolysaccharide biosynthesis proteins, lipid biosynthesis proteins, etc.

### Correlation between the metabolites of PNS and gut microbiota

**Fig. 5:**
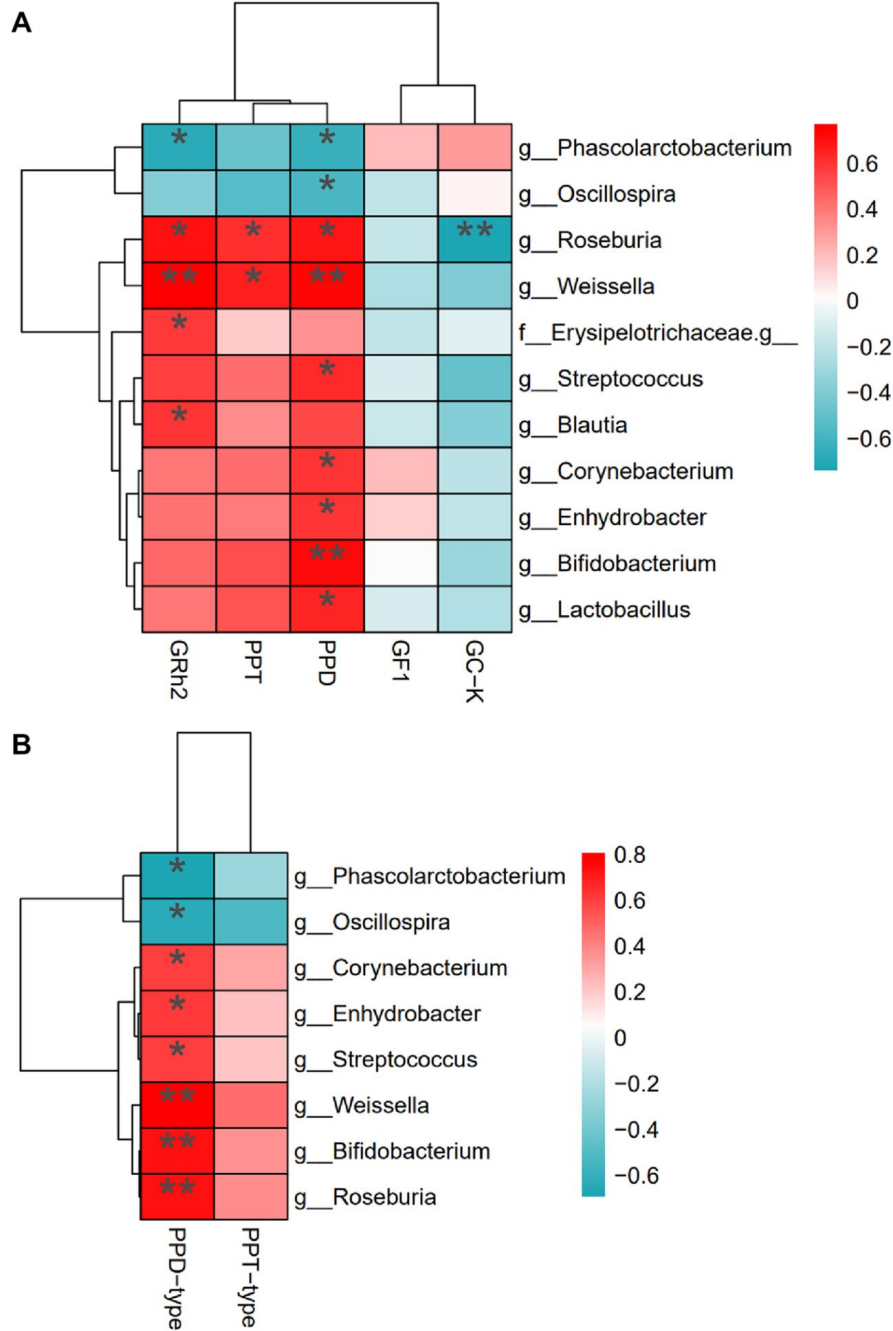
Heat map on Spearman’s correlations of main metabolites and Taxonomic differences of gut microbiota between LF-PF and HF-HP diet groups

Relative associations were analyzed using Spearman’s correlations index (Fig. 5A). *Corynebacterium*, *Enhydrobacter* and *Phascolarctobacterium* were positively associated with the yield of GF_1_, while *Blautia*, *Lactobacillus*, *Oscillospira*, *Roseburia*, *Streptococcus* and *Weissella* were inversely correlated with its abundance. GRh_2_ showed a positive association with the presence of *Blautia*, *Roseburia* and *Weissella*, while *Oscillospira* and *Phascolarctobacterium* were inversely correlated with its concentration. Strong positive correlations such as *Bifidobacterium*, *Corynebacterium*, *Enhydrobacter*, *Lactobacillus*, *Roseburia*, *Streptococcus* and *Weissella* with the yield of PPD were also confirmed. PPT had a significantly positive association with the presence of *Roseburia* and *Weissella*. Interestingly, compared with the metabolites of PPD-type ginsenosides, PPD-type ginsenosides indicated a stronger association with those gut microbials (Fig. 5B). In general, gut microbials enriched in the LF-PF group were positively correlated with PNS biotransformation. Herein, the data provided a meaningful link to understand the PNS metabolic differences mediated by personalized gut microbiota.

### Biotransformation of PNS by B. adolescentis and L. rhamnosus

**Fig. 6:**
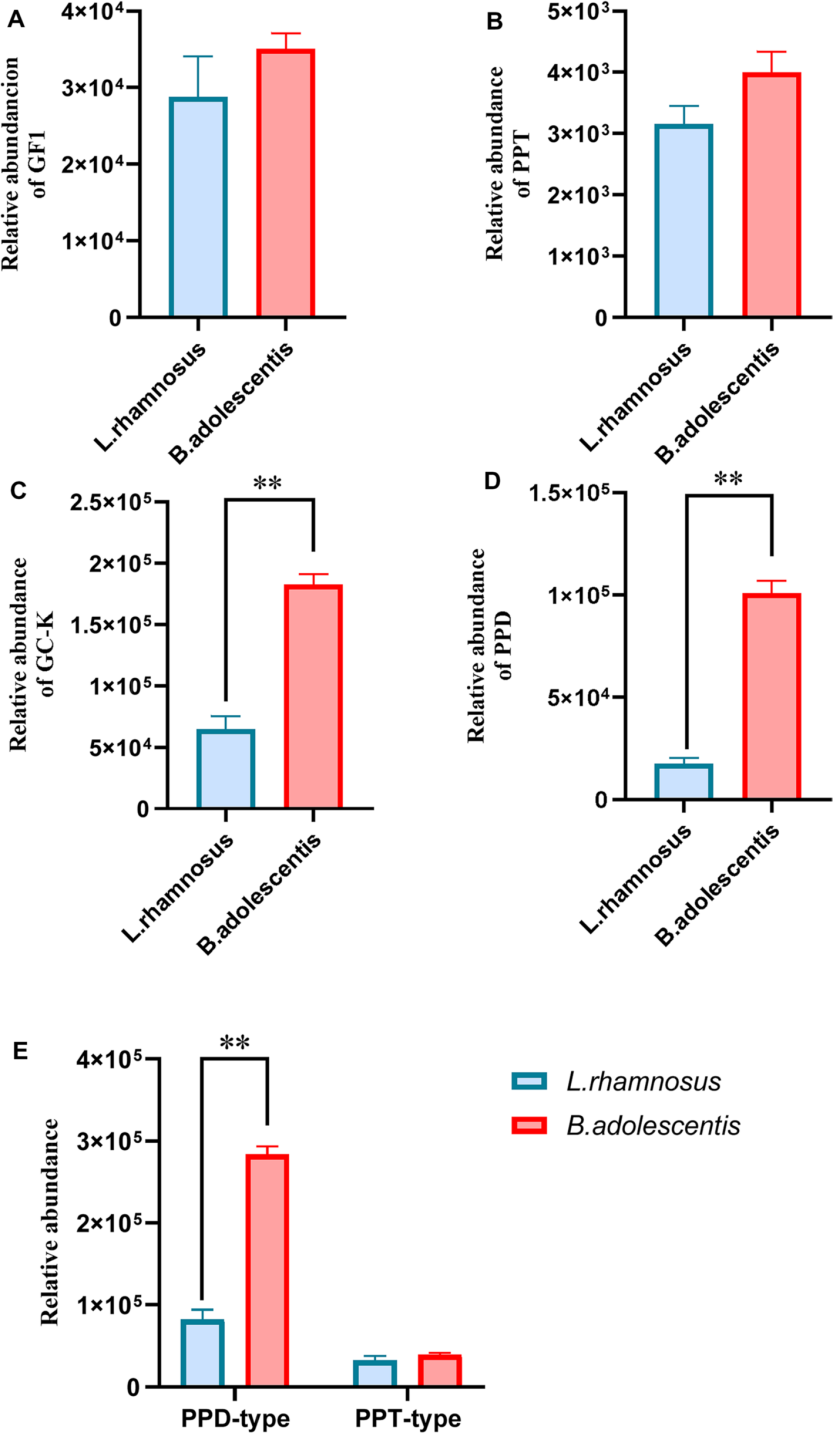
Relative quantities of main *P. notoginseng* saponins metabolites bio-converted by selected microbials. The relative quantities of GF_1_ (**A**), PPT (**B**), GC-K (**C**), PPD (**D**), PPT-type and PPD-type secondary ginsenosides (**E**) bio-transformed by *B. adolescentis* and *L. rhamnosus*, respectively

To confirm the metabolic capacity of specific bacteria species, *B. adolescentis* and *L. rhamnosus* were respectively incubated with PNS to evaluate the biotransformation. PNS were biotransformed to generate GF_1_, GC-K, PPD and PPT, while the metabolic profiles of PNS mediated by *B. adolescentis* and *L. rhamnosus* were different (Fig. 6). In general, *B. adolescentis* has a stronger ability to metabolize PNS than *L. rhamnosus*, especially PPD-type saponins, which implied that *B. adolescentis* might secrete *β*-glycosidases with stronger enzymatic catalysis.

## Discussion

In this paper, PNS could be metabolized by human gut microbiota. 16S rRNA gene sequencing technology was employed for analyzing the gut microbiota. Indeed, several metabolites have been reported as bioactive substances. GC-K is proved with potential anti-cancer effects through inducing cell apoptosis to inhibit tumor growth. Effects of GC-K on insulin resistance and abnormal vascular smooth muscle cell (VSMC) proliferation have also been evaluated [12, 13]. GC-K possess higher anti-proliferative effects on colon cancer than ginseng parent compounds, such as ginsenoside Rb_1_ (GRb_1_) [14]. Due to the increased hydrophobicity of GC-K, the absorption and distribution into tissue of GC-K is more easily than GRb_1_ [15]. In vivo, PNS were only catalyzed to generate GC-K by microbial *β*-glycosidases which could not be secreted from mammalian cells[5]. GRh_2_ possesses antineoplastic effects to inhibit metastasis of HepG2 liver carcinoma cell [16]. GF_1_ could prevent atherosclerosis by suppressing NF-*κ*B signaling pathway and down-regulating inflammatory factors expression [17]. PPT and PPD, as the metabolites of PPT- and PPD- type saponins, have low toxicity, relative good stability and potent biological activities, such as ameliorating glucose tolerance and insulin resistance [18]. PPD also plays important roles in redox equilibrium and neuroprotection through modulating the level of ROS or influencing mitochondrial function [19]. The beneficial biotransformation mediated by gut microbiota in human intestine plays an inevitable role to achieve pharmaceutical activities of PNS.

We have collected stool samples from LF-PF and HF-HP diet volunteers. The different diet-pattern healthy subjects are good candidates for strategies aiming at investigating different gut microbiota profiles. Our results are congruent with those of previous studies with decreased Firmicutes/Bacteroidetes (F/B) ratio in human gut microbiota driven by high-fat diet, while the low-fat diet increases abundance of *Blautia* and *Faecalibacterium* [8]. In addition, the abundance of *Bifidobacterium* and *Roseburia* in the high-fiber diet group are relatively higher [20], but the abundance of *Oscillospira* increases in the high-protein diet group [21]. Intriguingly, reduced diversity has been reported in gut microbiota of high-fat-fed mice [22], but our data showed relatively higher alpha-diversity in HF-HP group than LF-PF group. The inconsistency indicated healthy human gut microbiota were more complex than mice under controlled feeding condition. Analyzing the individual gut microbials in LF-PF group, the proportion of Firmicutes phyla was predominant in all phyla. Furthermore, the visualized PCoA showed two clusters to discriminate the inter-group variation. Alpha-diversity of gut microbials could be reshaped by diet patterns. Moreover, PNS could be hydrolyzed by *β*-glucosidases which is secreted by gut microbiota. Because *β*-glucosidase is differently secreted by specific gut microbial species, the yields of GRh_2_, PPT and PPD showed significantly differences between the two groups. GRh_2_, PPT and PPD were more easily metabolized by gut microbiota in HF-PF group. In addition, the abundances of PPD-type secondary ginsenosides were higher than PPT-type secondary ginsenosides. Interestingly, some specific gut microbials possess biotransformation preference pertinent to some stereochemical structures.

We explored the correlations between metabolic secondary ginsenosides and gut microbials. Interestingly, *Roseburia* and *Weissella* were significantly and positively correlated with the yields of PPD, PPT and GRh_2_, which had higher relative quantities in LF-PF group than HF-HP group. Furthermore, GRh_2_ showed a positive association with the presence of *Blautia*. Strong positive correlations were analyzed between *Bifidobacterium* and the yield of PPD, which was also positively correlated with *Corynebacterium*, *Enhydrobacter* and *Lactobacillus*. *Bifidobacterium* is able to uptake oligosaccharides for the fermentative metabolism of hexoses and pentoses [23]. *B. adolescentis* is selective for 4-nitrophenyl-*β*-D-glucopyranoside (pNPG) to 4-nitrophenyl-*β*-D-xylopyranoside (pNPX), with very low activity against other *β*, 1 → 4 and *β*, 1 → 2 substrates [24, 25]. Moreover, most low G + C% Gram-positive Firmicutes, *Blautia*, *Lactobacillus*, *Roseburia* and *Streptococcus*, have stronger *β*-glucosidases activity than other species [26]. Altogether, *Bifidobacterium*, *Blautia*, *Lactobacillus*, *Roseburia*, *Streptococcus* and *Weissella* enriched in LF-PF group may interpret the yield variations of GRh_2_, PPT and PPD between the two groups. The correlation between GC-K with the above-mentioned bacteria were negative, probably because GC-K could be further metabolized to be PPD. Furthermore, *Bifidobacterium, Roseburia* and *Weissella* showed stronger positive correlation with the PPD-type secondary ginsenosides. However, the accurate bacterial functions should be analyzed by metagenomic sequencing data in the future. Furthermore, due to an enterohepatic circulation of xenobiotics, it is also important to investigate PNS metabolic profile in vivo by systemically considering the gut microbiota and liver metabolism.

Consequently, the aim of this study was to investigate the bioconversion variations between PNS and gut microbials driven by two daily dietary patterns. Because of individual variations among each group, analysis should focus on the inter-group differences rather than consistency within the same group. Therefore, our study focused on the beta-diversity of gut microbiota between HF-HP and LF-PF groups, which led to the metabolic differences of PNS. Moreover, both of quality and quantity differences of PNS metabolites were also observed in two groups, which indicated gut microbiota diversity led to the metabolic differences of PNS. Depending on gut microbiota composition or function analysis, we could anticipate drug and drug-metabolite exposure for personalized adjustment to the dosage of medicine. However, detailed metagenomics and enlarged sample size should not be ignored to validate the relationship between specific microbial species and the yields of PNS metabolites.

## Conclusions

The bioconversion variations of PNS mediated by gut microbiota were observed to generate five main metabolites, including GF_1_, GRh_2_, GC-K, PPT and PPD, between LF-PF and HF-HP groups. The yields of GRh_2_, PPT and PPD in LF-PF group were much higher than HF-HP group. The profiles of gut microbiota between the two groups were significantly different, which indicated that the genus *Blautia*, *Bifidobacterium*, *Roseburia*, *Ruminococcus* and *SMB53*, enriched in LF-PF group, were positively correlated with PNS metabolites. PNS could be metabolized by *B. adolescentis* and *L. rhamnosus* to generate the above-mentioned metabolites.

## Supplementary Information

**Additional file 1: Figure S1.** Chemical structures of *P. notoginseng* saponins metabolites. **Figure S2.** Typical MRM chromatograms of blank samples (a) and blank samples spiked with IS or with analytes (b) in positive ion mode. b1, b2, b3, b4, b5 and b6 were typical MRM chromatograms of blank samples spiked with GF_1_, PPT, GRh_2_, GC-K, PPD or IS Digoxin, and a1, a2, a3, a4, a5 and a6 were the chromatograms of blank samples. **Figure S3.** Mass spectra and fragmentation pathways of GF_1_ (a), PPT (b), GRh_2_ (c), GC-K (d), PPD (e) and Digoxin (f) in positive ion mode. **Figure S4.** Typical TICs of mixed standards (including GF_1_, PPT, GRh_2_, GC-K, PPD and Digoxin) (a) and PNS metabolites bio-converted by gut microbiota collected from LF-PF (b) and HF-HP (c) diet groups at 37 ℃for 0 h and 48 h in positive ion mode. **Figure S5.** Rarefaction curve based on Shannon index (a) and observed OTU numbers (b). **Figure S6.** Differences in KEGG pathway enrichment between HF-HP and LF-PF groups. **Figure S7.** The effects of PNS on the growth dynamics of *L. rhamnosus* (a) and *B. adolescentis* (b).

**Additional file 2: Table S1.** MRM parameters of detected compounds. **Table S2.** Precision of five main metabolites (mean, RSD < 15%). **Table S3.** Energy content, macronutrient composition, and fiber content of the HF-HP and LF-PF diets.

## Data Availability

The original data generated from this study are accompanied with the article as additional files.

## Abbreviations

16S rRNA: 16S ribosomal RNA
DH-PPT: Didehydro-protopanaxatriol
DMSO: Dimethyl sulphoxide
F/B ratio: The Firmicutes/Bacteroidetes ratio
GAM: General anaerobic medium
GC-K: Ginsenoside compound K
GF_1_: Ginsenoside F_1_
GRb_1_: Ginsenoside Rb_1_
GRh_2_: Ginsenoside Rh_2_
HF-HP diet: High-fat and high-protein diet
HPLC–ESI–MS/MS: A high performance liquid chromatography-electrospray ionization tandem mass spectrometry
LDA: Linear discriminative analysis
LEfSe: The linear discriminative analysis effect size
LF-PF diet: Low-fat and plant fiber-rich diet
OTUs: Operational taxonomic units
PCoA: Principal component analysis
PC: Principal component
PCR: Polymerase Chain Reaction
PNS: *Panax notoginseng* Saponins
PPD: Protopanaxadiol
PPT: Protopanaxatriol
QIIME: Quantitative Insights Into Microbial Ecology
TCMs: Traditional Chinese medicines
VSMC: Vascular smooth muscle cell
