# Supplementary material for: Personalized bioconversion of Panax notoginseng saponins mediated by gut microbiota between two different diet-pattern healthy subjects
Source: Chin Med. 2021 Jul 23;16:60. doi: 10.1186/s13020-021-00476-5 (PMC8306348; doi:10.1186/s13020-021-00476-5)
Supplement: Supplementary file 1 — Additional file 1: Figure S1. Chemical structures of P. notoginseng saponins metabolites. Figure S2. Typical MRM chromatograms of blank samples (a) and blank samples spiked with IS or with analytes (b) in positive ion mode. b1, b2, b3, b4, b5 and b6 were typical MRM chromatograms of blank samples spiked with GF1, PPT, GRh2, GC-K, PPD or IS Digoxin, and a1, a2, a3, a4, a5 and a6 were the chromatograms of blank samples. Figure S3. Mass spectra and fragmentation pathways of GF1 (a), PPT (b), GRh2 (c), GC-K (d), PPD (e) and Digoxin (f) in positive ion mode. Figure S4. Typical TICs of mixed standards (including GF1, PPT, GRh2, GC-K, PPD and Digoxin) (a) and PNS metabolites bio-converted by gut microbiota collected from LF-PF (b) and HF-HP (c) diet groups at 37 ℃for 0 h and 48 h in positive ion mode. Figure S5. Rarefaction curve based on Shannon index (a) and observed OTU numbers (b). Figure S6. Differences in KEGG pathway enrichment between HF-HP and LF-PF groups. Figure S7. The effects of PNS on the growth dynamics of L. rhamnosus (a) and B. adolescentis (b). [file 13020_2021_476_MOESM1_ESM.pptx]

## Slide 1
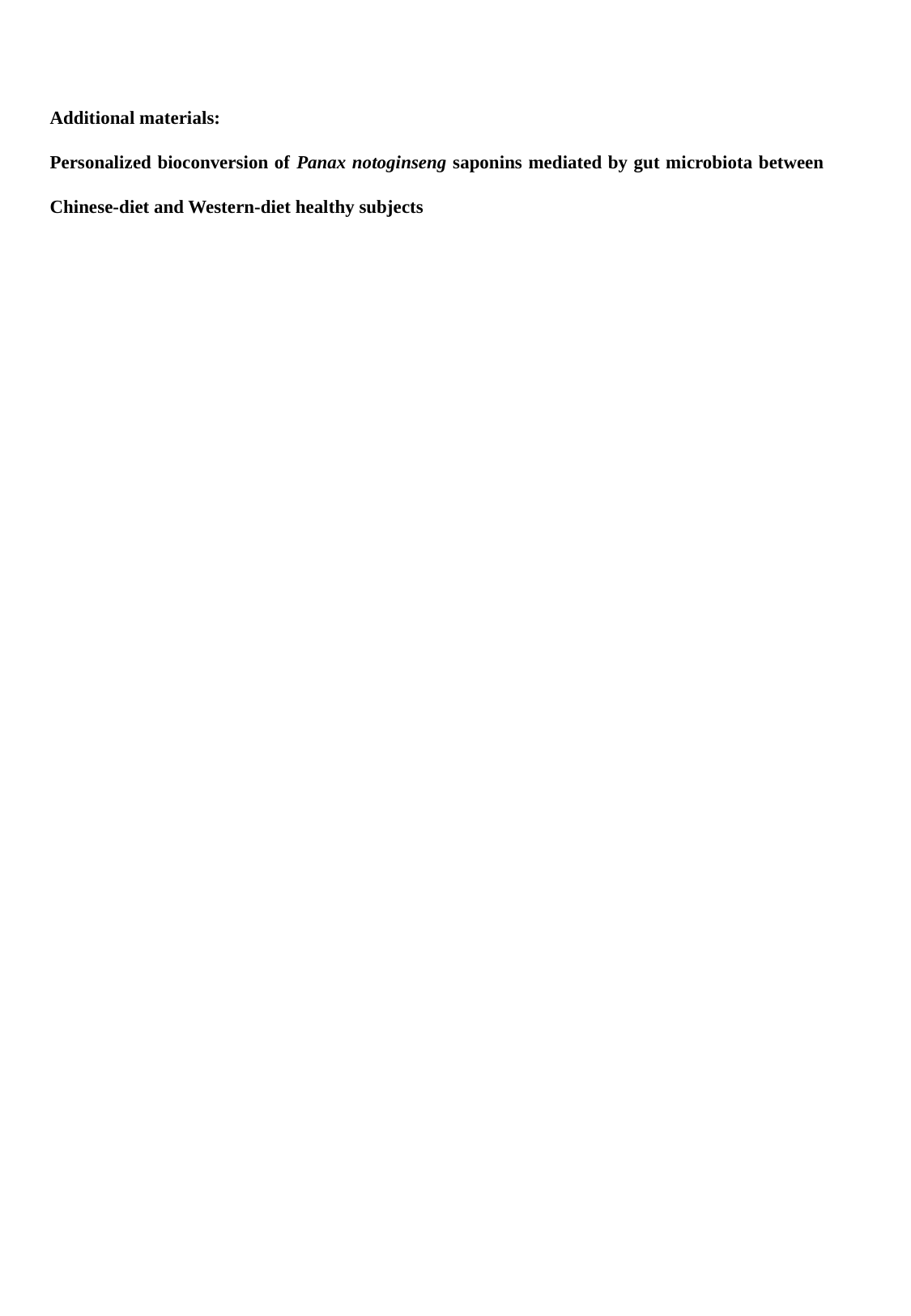

Additional materials:
Personalized bioconversion of Panax notoginseng saponins mediated by gut microbiota between Chinese-diet and Western-diet healthy subjects

## Slide 2
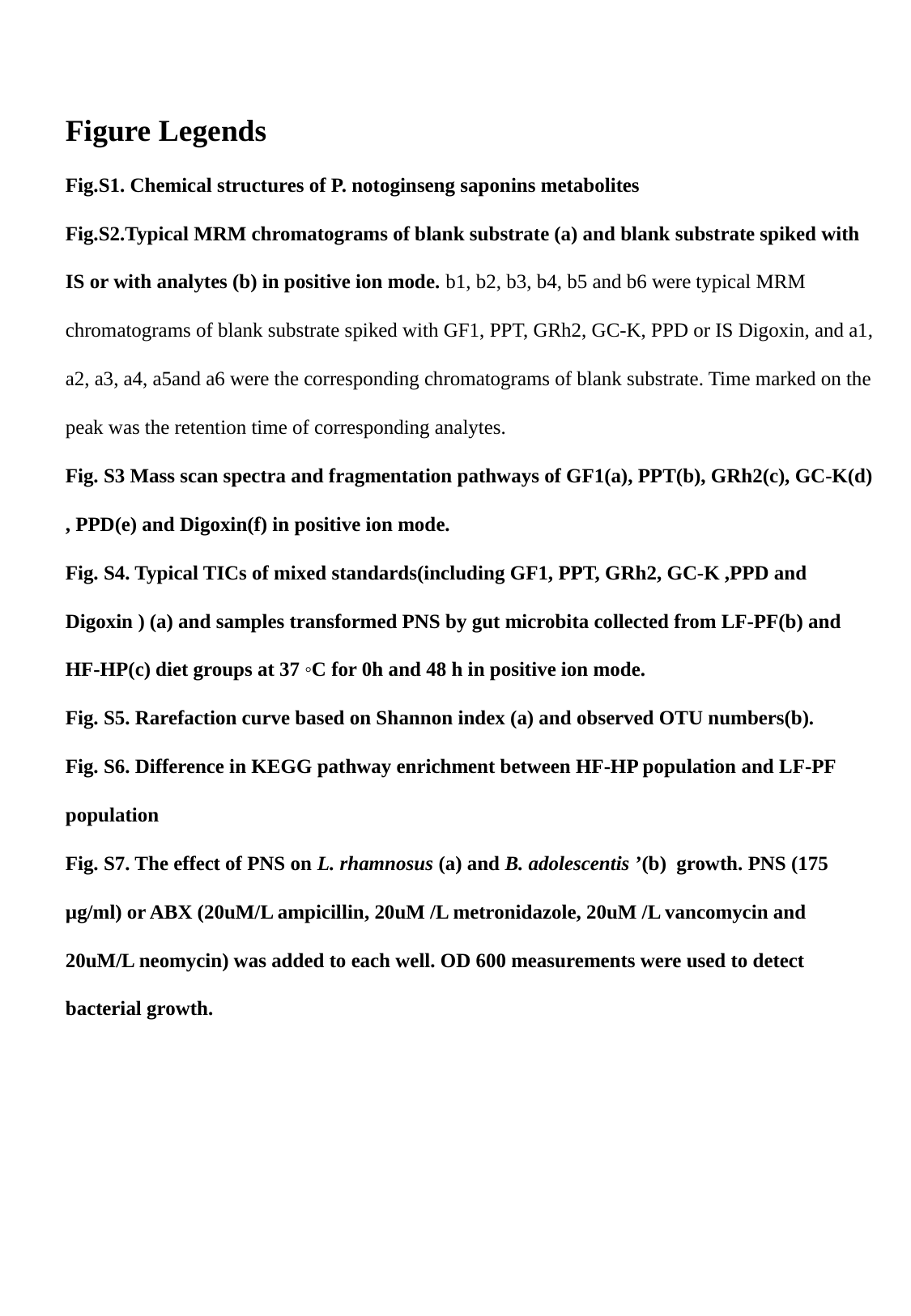

Figure Legends
Fig.S1. Chemical structures of P. notoginseng saponins metabolites
Fig.S2.Typical MRM chromatograms of blank substrate (a) and blank substrate spiked with IS or with analytes (b) in positive ion mode. b1, b2, b3, b4, b5 and b6 were typical MRM chromatograms of blank substrate spiked with GF1, PPT, GRh2, GC-K, PPD or IS Digoxin, and a1, a2, a3, a4, a5and a6 were the corresponding chromatograms of blank substrate. Time marked on the peak was the retention time of corresponding analytes.
Fig. S3 Mass scan spectra and fragmentation pathways of GF1(a), PPT(b), GRh2(c), GC-K(d) , PPD(e) and Digoxin(f) in positive ion mode.
Fig. S4. Typical TICs of mixed standards(including GF1, PPT, GRh2, GC-K ,PPD and Digoxin ) (a) and samples transformed PNS by gut microbita collected from LF-PF(b) and HF-HP(c) diet groups at 37 ◦C for 0h and 48 h in positive ion mode.
Fig. S5. Rarefaction curve based on Shannon index (a) and observed OTU numbers(b).
Fig. S6. Difference in KEGG pathway enrichment between HF-HP population and LF-PF population
Fig. S7. The effect of PNS on L. rhamnosus (a) and B. adolescentis ’(b) growth. PNS (175 µg/ml) or ABX (20uM/L ampicillin, 20uM /L metronidazole, 20uM /L vancomycin and 20uM/L neomycin) was added to each well. OD 600 measurements were used to detect bacterial growth.

## Slide 3
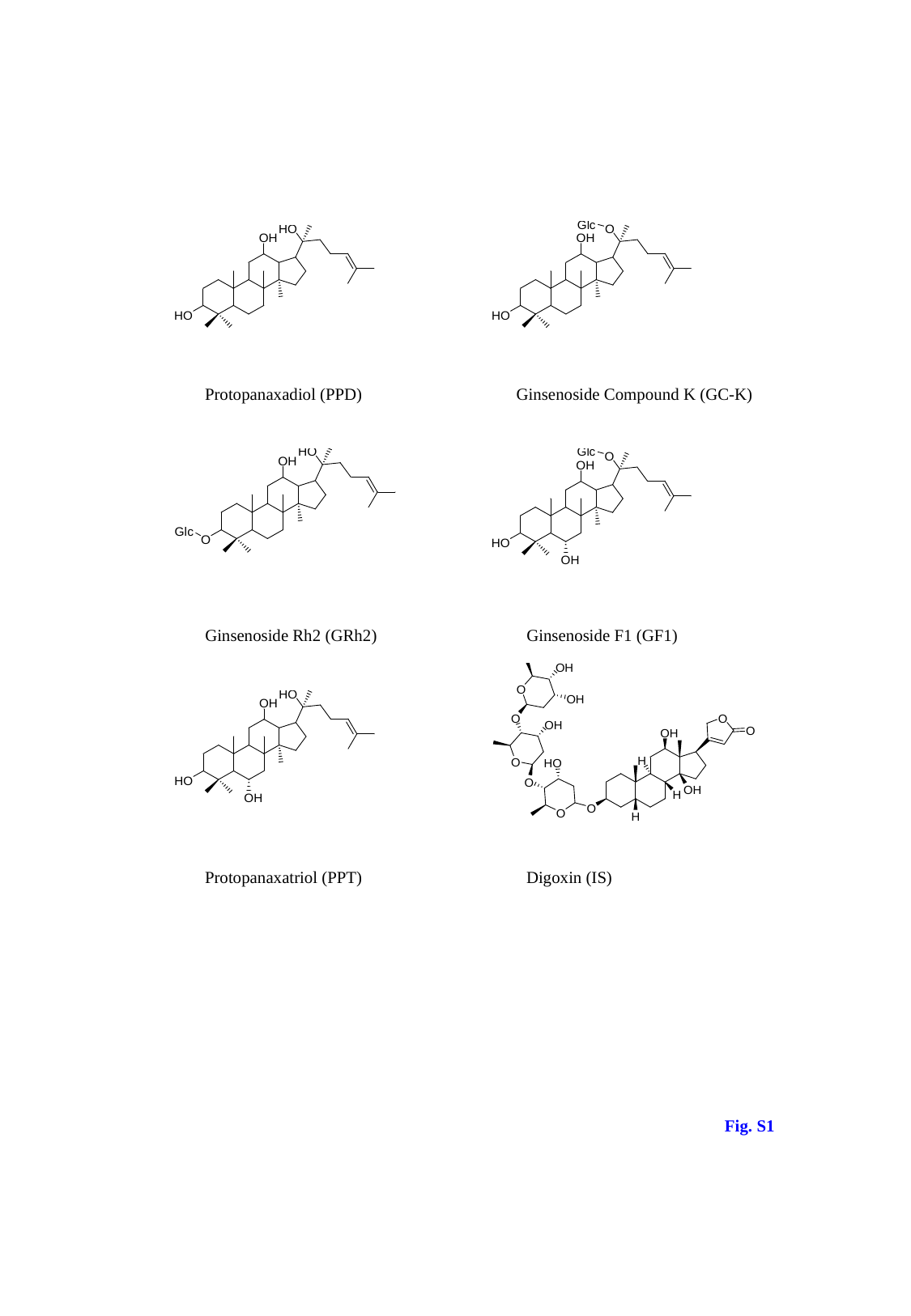

Protopanaxadiol (PPD)
Ginsenoside Compound K (GC-K)
Ginsenoside Rh2 (GRh2)
Ginsenoside F1 (GF1)
Protopanaxatriol (PPT)
Digoxin (IS)
Fig. S1

## Slide 4
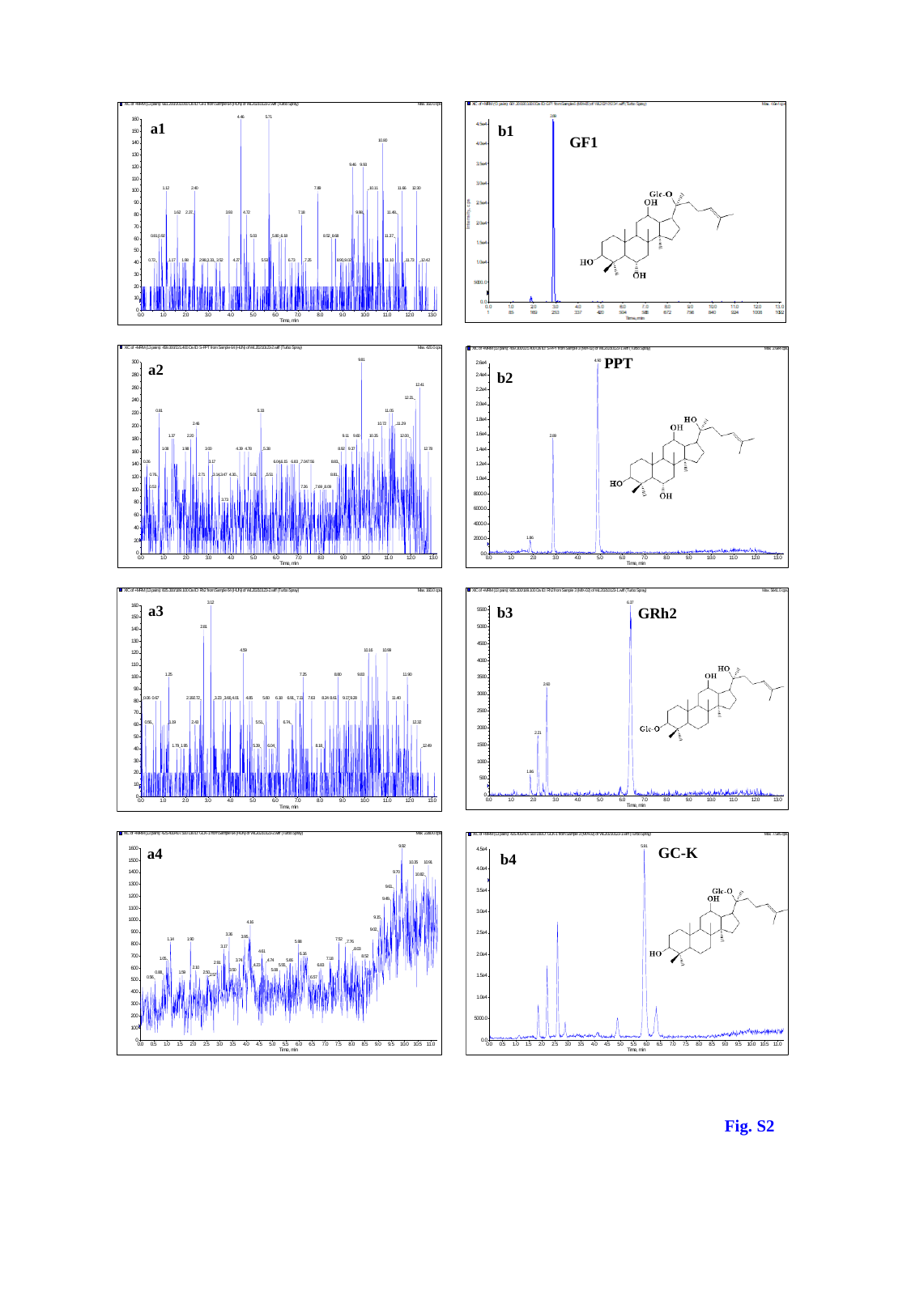

a1
b1
GF1
PPT
a2
b2
a3
b3
GRh2
a4
GC-K
b4
Fig. S2

## Slide 5
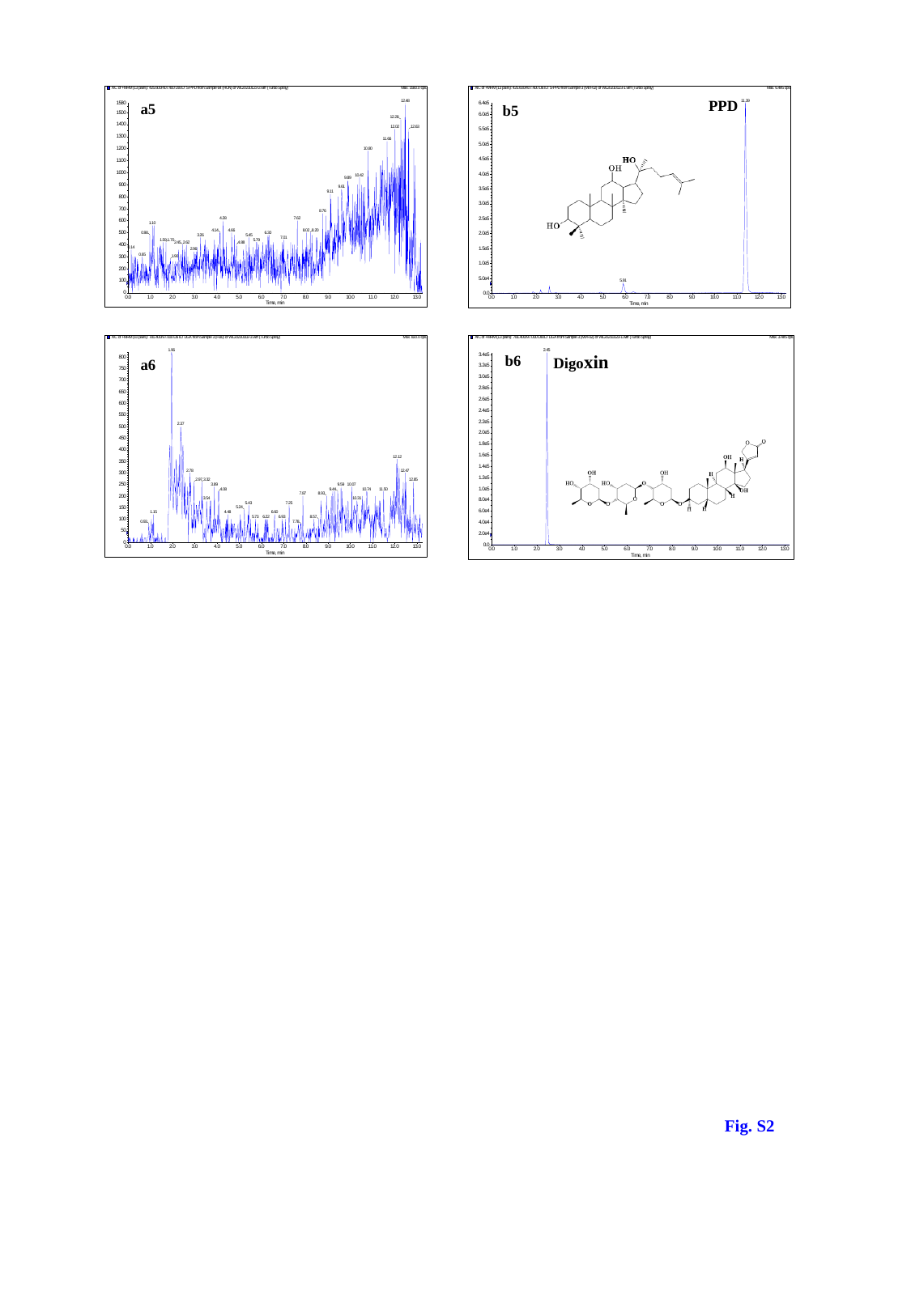

PPD
a5
b5
Digoxin
b6
a6
Fig. S2

## Slide 6
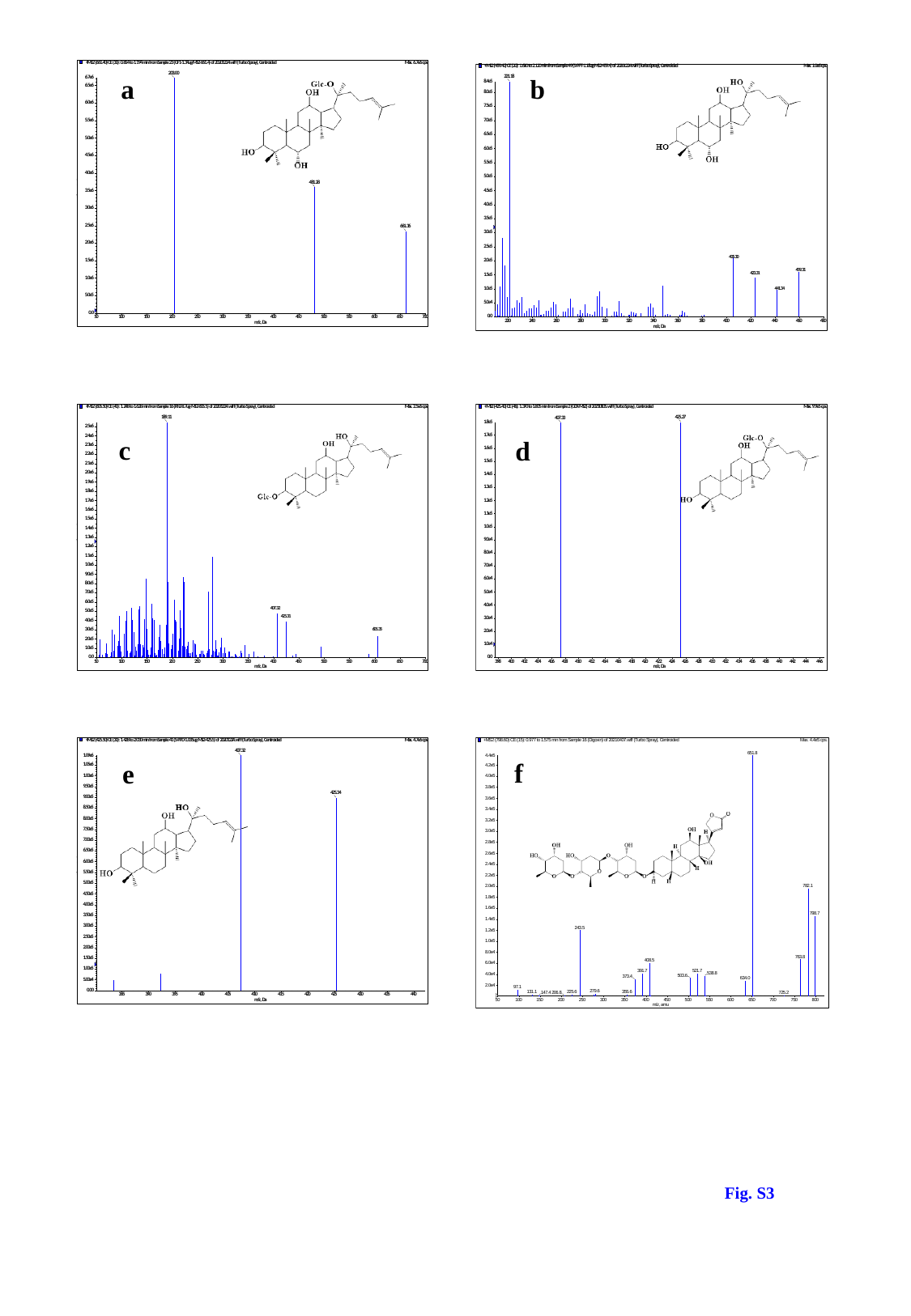

a
b
c
d
f
e
Fig. S3

## Slide 7
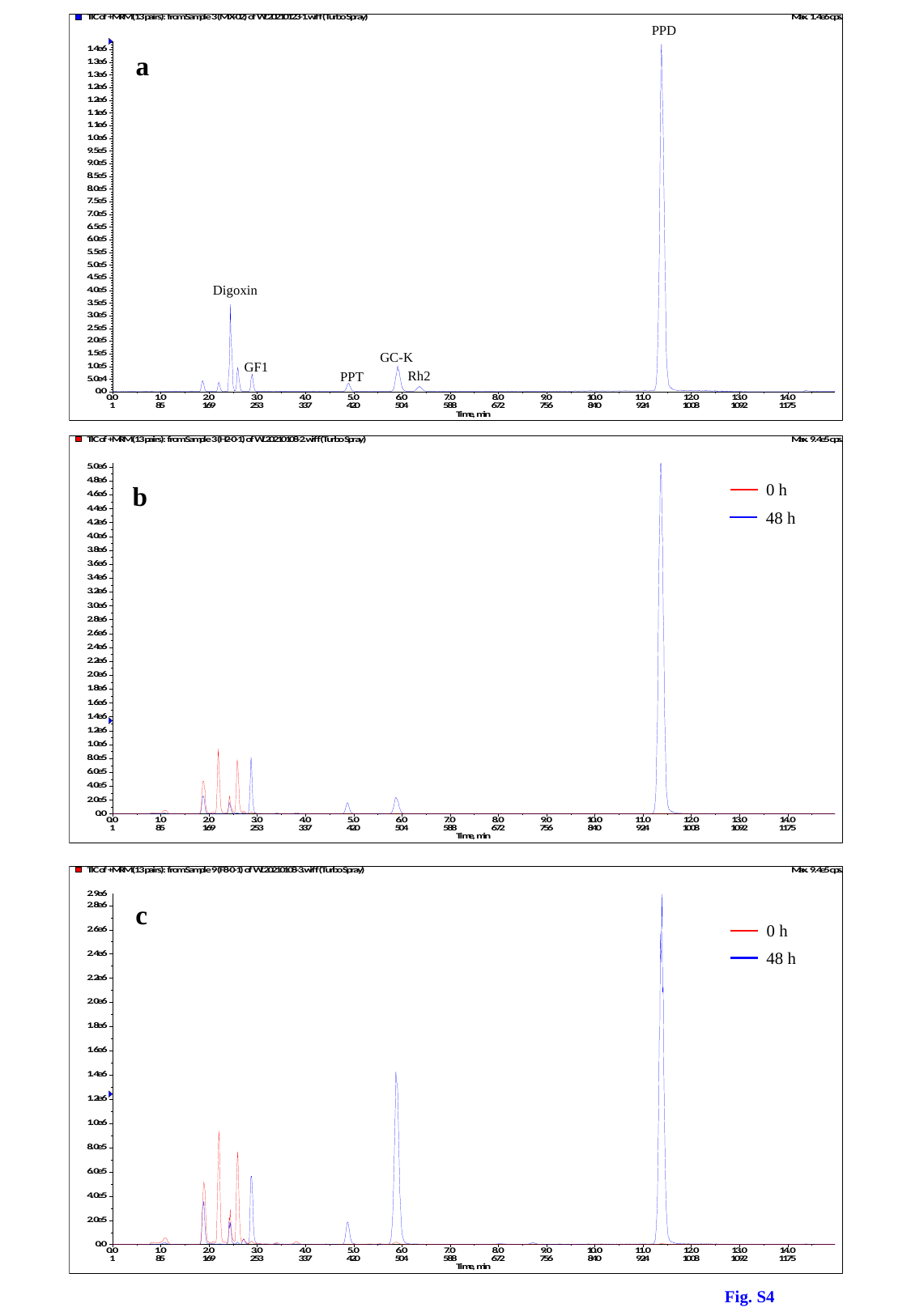

PPD
a
Digoxin
GC-K
GF1
Rh2
PPT
b
0 h
48 h
c
0 h
48 h
Fig. S4

## Slide 8
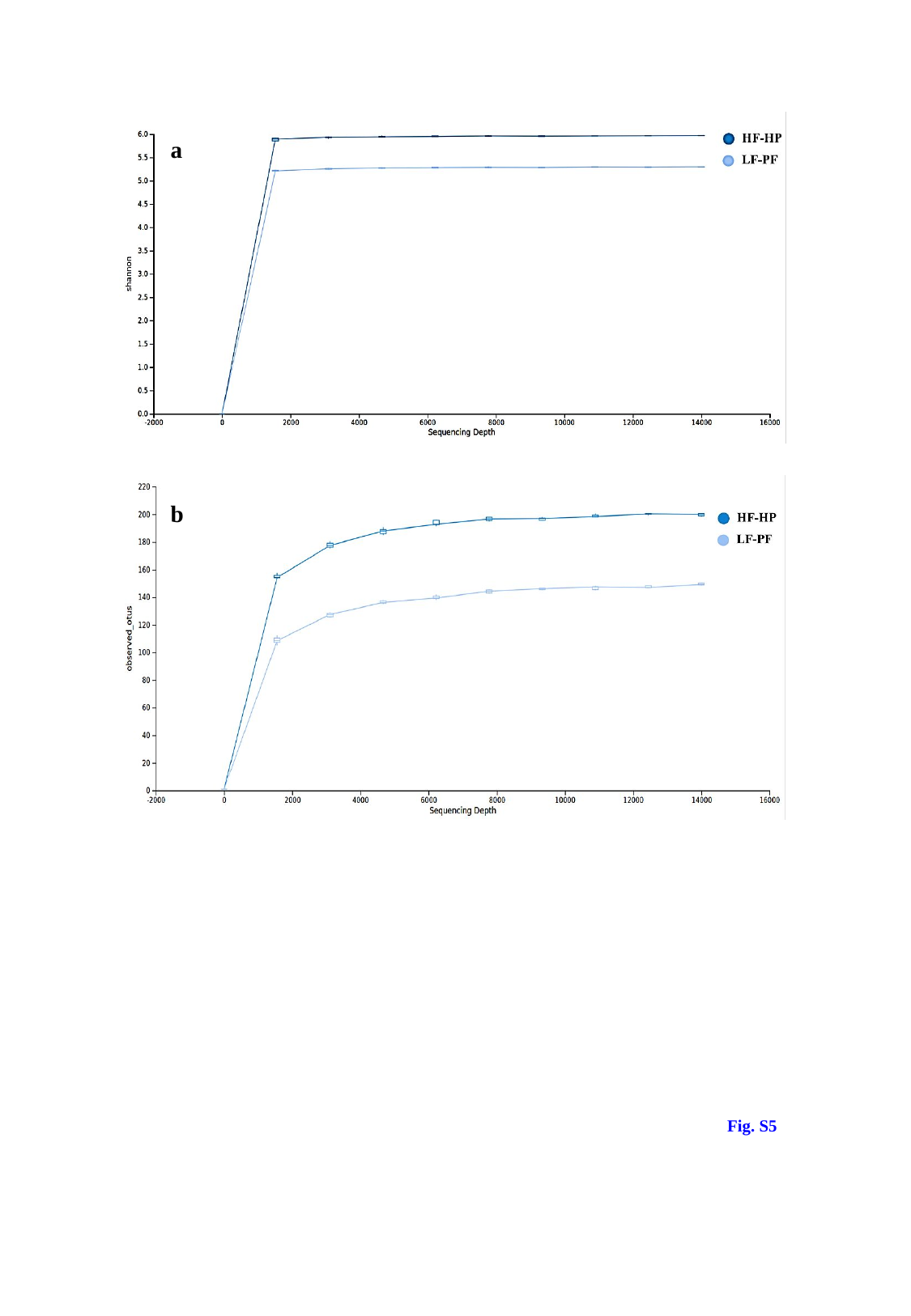

a
b
Fig. S5

## Slide 9
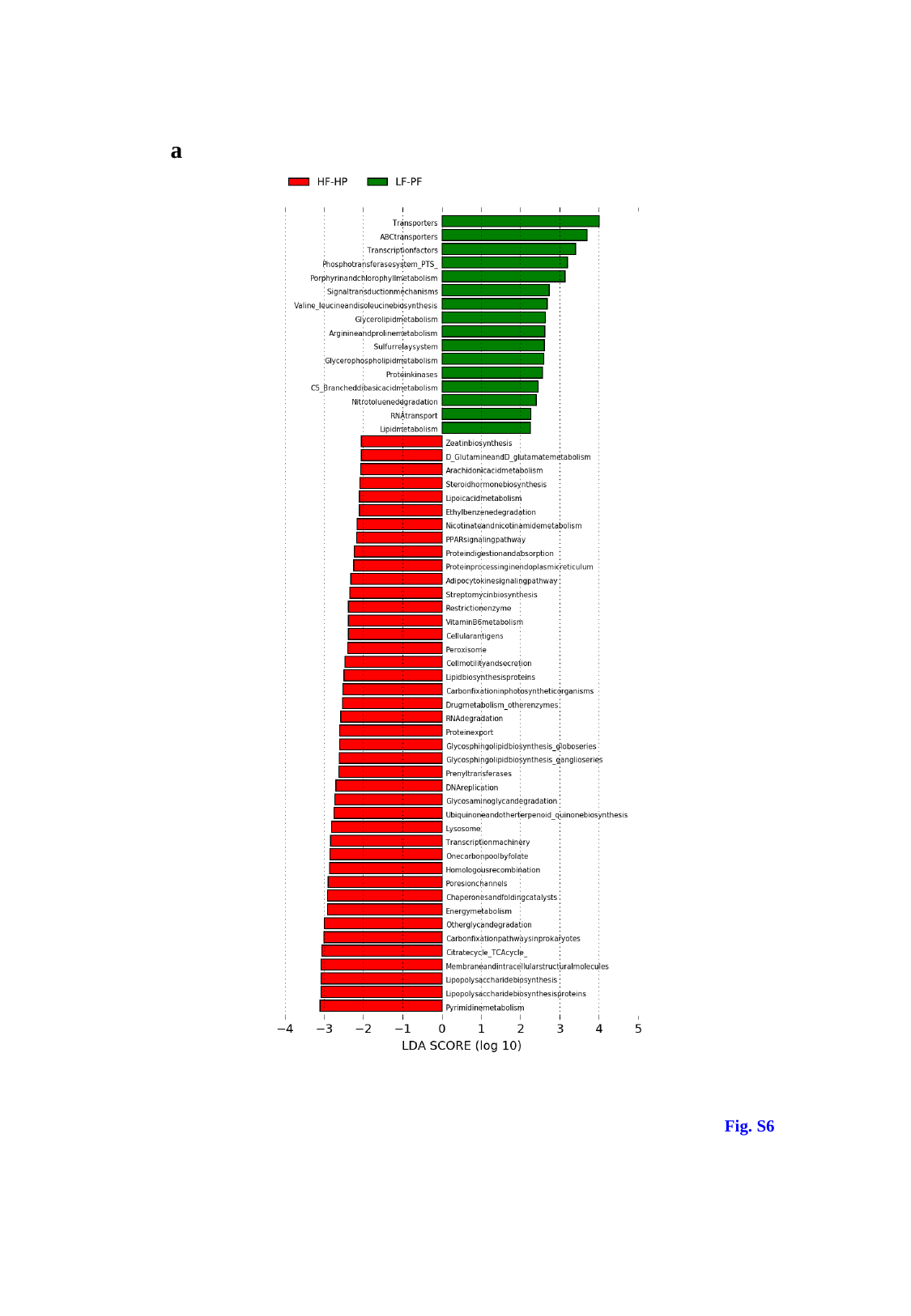

a
Fig. S6

## Slide 10
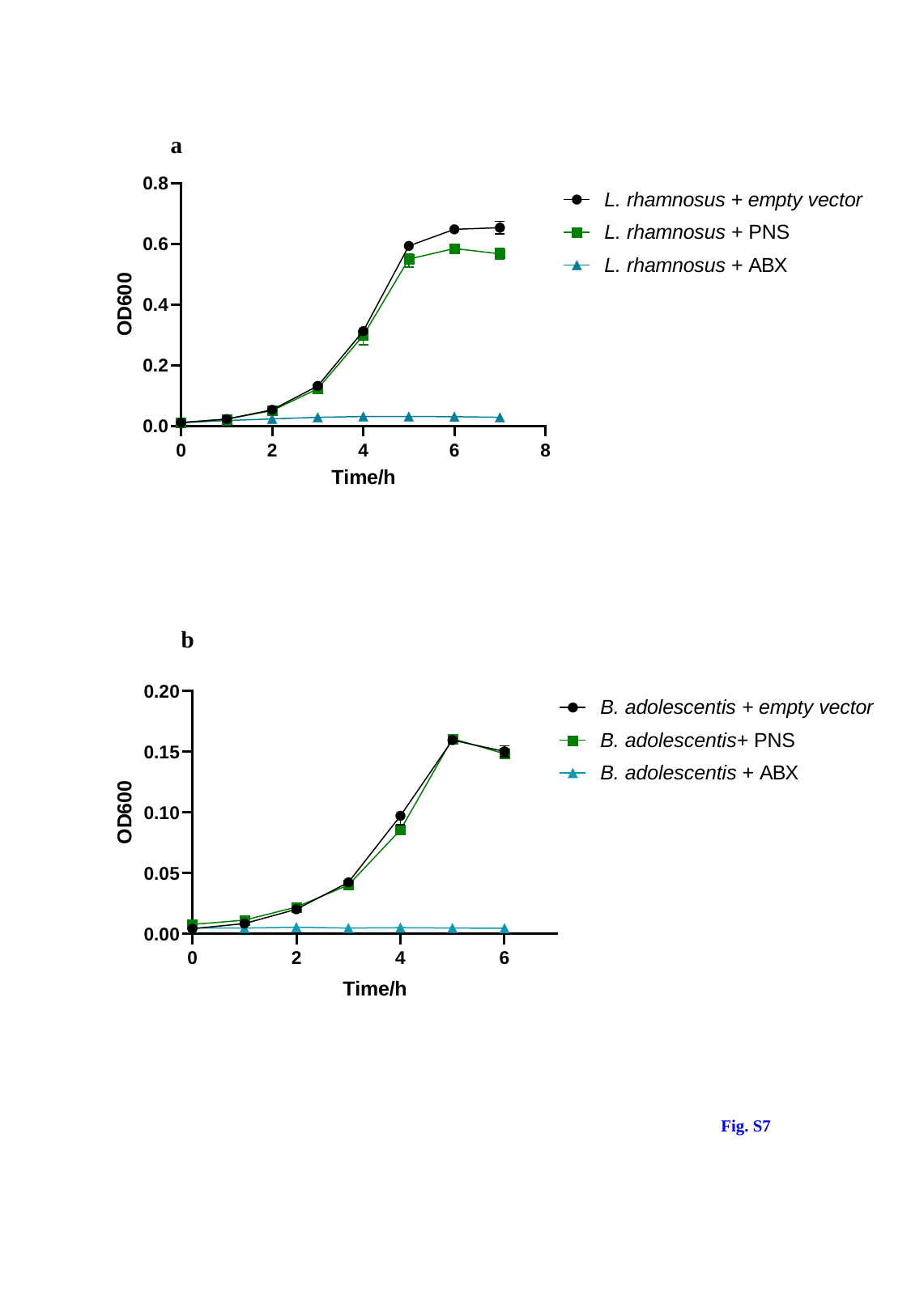

a
b
Fig. S7
