# Supplementary material for: Personalized bioconversion of Panax notoginseng saponins mediated by gut microbiota between two different diet-pattern healthy subjects
Source: Chin Med. 2021 Jul 23;16:60. doi: 10.1186/s13020-021-00476-5 (PMC8306348; doi:10.1186/s13020-021-00476-5)
Supplement: Supplementary file 2 — Additional file 2: Table S1. MRM parameters of detected compounds. Table S2. Precision of five main metabolites (mean, RSD < 15%). Table S3. Energy content, macronutrient composition, and fiber content of the HF-HP and LF-PF diets. [file 13020_2021_476_MOESM2_ESM.docx]

**Additional Table**

**Personalized bioconversion of *Panax notoginseng* saponins mediated by gut microbiota between Chinese-diet and Western-diet healthy subjects**

Additional file 1: Table S1 MRM parameters of detected compounds

| Compounds | Q1/Q3(Da) | Dwell Time(ms) | CE(V) | DP(V) | EP(V) | CXP(V) |
| --- | --- | --- | --- | --- | --- | --- |
| GCK | 425.4/407.5 | 100 | 21 | 92 | 9 | 8 |
| GRh_2_ | 605.3/198.1 | 100 | 42 | 88 | 10 | 10 |
| GF_1_ | 661.2/203.0 | 100 | 45 | 147 | 6 | 11 |
| PPD | 425.6/407.4 | 100 | 22 | 72 | 7 | 13 |
| PPT | 459.3/221.4 | 100 | 36 | 124 | 7 | 20 |
| Digoxin | 781.4/97.0 | 100 | 50 | 90 | 8 | 9 |

Additional file 1: Table S2 Precision of five main metabolites(mean, RSD＜15%)

| **Compounds** | **Conc. added (ng/mL)** | | **Intra-day (n = 6)** | **Inter-day (n=18)** |
| --- | --- | --- | --- | --- |
|  |  |  | **Precision (%)** | **Precision (%)** |
| **GCK** | 14.8 | 6.42 | | 12.1 |
| **RH_2_** | 11.9 | 6.67 | | 13.3 |
| **GF1** | 13.1 | 8.77 | | 13.4 |
| **PPD** | 14.9 | 8.37 | | 13.3 |
| **PPT** | 96.7 | 8.63 | | 12.9 |

Additional file 1: Table S3. Energy content, macronutrient composition, and fiber content of the HF-HP and LF-PF diets

|  | HF-HP diet | LF-PF diet |
| --- | --- | --- |
| Protein （% of energy) | 20~ | ~10 |
| Carbohydrates （% of energy) | ~50 | ~60 |
| Fat （% of energy) | 35~40 | ~20 |
| Fiber (g/day) | ~5 | 20~ |
